# Patterns of Migration and Risks Associated with Leprosy among Migrants in Maranhão, Brazil

**DOI:** 10.1371/journal.pntd.0002422

**Published:** 2013-09-05

**Authors:** Christine Murto, Frédérique Chammartin, Karolin Schwarz, Lea Marcia Melo da Costa, Charles Kaplan, Jorg Heukelbach

**Affiliations:** 1 Swiss Tropical and Public Health Institute, Basel, Switzerland; 2 University of Basel, Basel, Switzerland; 3 University of Cologne, Cologne, Germany; 4 Leprosy Control Program; State Health Secretariat of Maranhão, São Luis, Brazil; 5 University of Southern California, School of Social Work, Hamovitch Center for Science in the Human Services, Los Angeles, California, United States of America; 6 Federal University of Ceará, Fortaleza, Brazil; 7 Anton Breinl Centre for Public Health and Tropical Medicine, James Cook University, Townsville, Australia; Kwame Nkrumah University of Science and Technology (KNUST) School of Medical Sciences, Ghana

## Abstract

Leprosy remains a public health problem in Brazil with new case incidence exceeding World Health Organization (WHO) goals in endemic clusters throughout the country. Migration can facilitate movement of disease between endemic and non-endemic areas, and has been considered a possible factor in continued leprosy incidence in Brazil. A study was conducted to investigate migration as a risk factor for leprosy. The study had three aims: (1) examine past five year migration as a risk factor for leprosy, (2) describe and compare geographic and temporal patterns of migration among past 5-year migrants with leprosy and a control group, and (3) examine social determinants of health associated with leprosy among past 5-year migrants. The study implemented a matched case-control design and analysis comparing individuals newly diagnosed with leprosy (n = 340) and a clinically unapparent control group (n = 340) without clinical signs of leprosy, matched for age, sex and location in four endemic municipalities in the state of Maranhão, northeastern Brazil. Fishers exact test was used to conduct bivariate analyses. A multivariate logistic regression analysis was employed to control for possible confounding variables. Eighty cases (23.5%) migrated 5-years prior to diagnosis, and 55 controls (16.2%) migrated 5-years prior to the corresponding case diagnosis. Past 5 year migration was found to be associated with leprosy (OR: 1.59; 95% CI 1.07–2.38; p = 0.02), and remained significantly associated with leprosy after controlling for leprosy contact in the family, household, and family/household contact. Poverty, as well as leprosy contact in the family, household and other leprosy contact, was associated with leprosy among past 5-year migrants in the bivariate analysis. Alcohol consumption was also associated with leprosy, a relevant risk factor in susceptibility to infection that should be explored in future research. Our findings provide insight into patterns of migration to localize focused control efforts in endemic areas with high population mobility.

## Author Summary

In Brazil, leprosy remains a significant public health problem in endemic clusters of high transmission risk throughout the country. Migration is thought to be a factor associated with continued leprosy transmission, as migration has also been found to be associated with other Neglected Tropical Diseases (NTDs). We analyzed the association between past five year migration and leprosy as part of a larger epidemiological study evaluating risk factors for infection among recently diagnosed leprosy cases (n = 340) and a matched clinically unapparent control group (n = 340) in the northeastern state of Maranhão. Among migrants with leprosy, 23.5% (n = 80) migrated in the past five years, with 16.2% (n = 55) of the control group. Past five year migration was significantly associated with leprosy, and remained significant after controlling for household and familial contact as potential confounders. Factors found to be associated with leprosy among past 5-year migrants included alcohol consumption, poverty, and household, family and other leprosy contact. Key patterns of movement emerged from the study that may aid future regional leprosy control efforts.

## Introduction

Leprosy continues to be an endemic disease in many parts of the world. Brazil has globally the second highest new case incidence [1]. National leprosy prevalence of 1.54/10,000 in 2010 [2] remains above the WHO goal of <1 per 10,000. Highly endemic areas of the disease continue to persist despite large-scale national efforts to control the disease. A challenge in disease control efforts is compounded as leprosy can be diagnosed many years after infection took place due to the long incubation period, and mild early symptoms of the disease may be overlooked. Migration has been found to be a social determinant of disease [3], and has been hypothesized as a risk factor in continued leprosy incidence [4], [5], [6]. In fact, earlier research in Brazil highlighted the increased distribution of leprosy along new corridors coinciding with frontier expansion connecting southern agricultural areas to the north of Brazil [7], as well as periurban migrant settlements on the outskirts of urban centers [4]. Migrants move between endemic and non-endemic areas in Brazil and often live in substandard conditions. As an infectious disease caused by *Mycobacterium leprae*, leprosy primarily affects the skin and peripheral nerves and causing sensory loss. While nasal mucosa is considered the main transmission site, new research indicates that oral presence of *M.leprae* bacilli may be an additional mode of transmission [8]. Maranhão, the study area of this research, has the third highest prevalence of leprosy (5.34/10,000) in the country [2] and is among the states with the highest out- and return- migration rates [9].

The proliferation of leprosy in Brazil continues largely in conditions of poverty that include poor housing and sanitation, high household density, illiteracy and low socioeconomic levels both at the micro and macro levels [4], [10]–[13]. Rapid population growth and uncontrolled urbanization, often as a consequence of migration for employment and differential access to services between rural and urban areas, has facilitated the expansion of these poor social and environmental conditions on the peripheries of cities associated with leprosy infection [4]–[5], [7], [13]. Additionally, new road construction and railways have enabled movement between rural communities and urban areas. These developments in transportation have been argued to explain the expanded distribution of leprosy in Brazil [4]–[6]. Nevertheless, household leprosy contact continues to be the primary risk factor associated with leprosy infection [14]. Proximity to the household contact has been seen as relevant in terms of increased risk [15]. Consanguineous contact has also been found to be associated with leprosy. Findings from Moet et al. (2006) suggest evidence of a genetic relationship independent of physical contact for leprosy infection.

Migration has been found to be an impediment to both leprosy elimination and control efforts. Prior research has suggested that migration may influence transmission and distribution of the disease [5], [16] as well as other neglected tropical diseases (NTDs) [3], [17]–[23]. This study explores the spatial and temporal patterns of migration in individuals with leprosy in Maranhão. The study also examines risk factors associated with leprosy among individuals who have migrated in the past five years (past 5-year migrants). Comparison of risks associated with leprosy and migration is challenging in a homogeneous population. However evaluation of specific risk factors that differentiate leprosy among past 5-year migrants from a clinically unapparent control group without clinical signs of leprosy who migrated in the past five years in this investigation, sheds light on those factors that are of importance when considering leprosy infection and expression of disease. The study has three specific aims: 1) to examine if migration in the past five years is a risk factor for leprosy; 2) to describe and compare geographic and temporal patterns of migration among past 5-year migrants with leprosy and a control group without clinical signs of leprosy; 3) to examine the social determinants of health associated with leprosy among past 5-year migrants.

## Methods

### Ethics statement

Written approval was obtained from the Ethical Review Board of the Federal University of Ceará (Fortaleza, Brazil). Permission to perform the study was also obtained by the Maranhão State Health Secretariat, the State Leprosy Control Program and municipalities involved. Informed written consent was obtained from study participants, or their parent/guardian in the case of minors, after explaining the objectives of the study. Interviews were conducted in private.

### Study area

The research was conducted in four leprosy endemic municipalities in the state of Maranhão, Brazil: Santa Inês, São José de Ribamar, Codó, and Bacabal. These municipalities are located in a major endemic cluster identified by the Brazilian Ministry of Health as a high-risk area for leprosy transmission [16]. Santa Inês, (population 77,282) [24], Codó (population 118,038) [24], and Bacabal (population 100,014 same) [24] are small townships in the interior of Maranhão that are largely surrounded by rural agricultural production, while São José de Ribamar (population 163,045) [24] is on the outskirts of the capital city, São Luis. Most households are small brick or mud and palm residences with rudimentary plumbing and hammocks to accommodate the multigenerational inhabitants.

### Study design

A case-control study was designed as part of an extended epidemiological investigation on risk factors associated with leprosy infection in four highly endemic municipalities in Maranhão, as part of the MAPATOPI study. The MAPATOPI study is an interdisciplinary project to support and improve the Brazilian leprosy program in Maranhão, Pará, Tocantins, and Piaui. Variables associated with past five year migration among those diagnosed with leprosy between 2009–2010 were compared with a matched clinically unapparent control group without clinical signs of leprosy. Migration was defined as those who resided outside of the municipality of their current residence, and is limited to five years as this is the average incubation period from leprosy infection to symptom onset. Past five year migration data is also collected in the Brazilian National Household Survey [9]. A detailed analysis of socio-cultural, health service related and economic variables that were collected as part of the larger epidemiological study will be explored elsewhere.

### Study sample

The case group was identified through the database of the National Information System for Notifiable Diseases (*Sistema de Informação de Agravos de Notificação* – SINAN) and included adults 15 and older in each of the four sites diagnosed with leprosy in 2009–2010 (n = 394). Individuals under 15 years of age, those previously diagnosed with leprosy and relapsed, living outside of the highly endemic cluster and who could not be located through multiple contact attempts were excluded from the study. The control group (n = 391) was selected from the *Programa Saúde da Família* (Program for Family Health). This program registers all families in the catchment areas of the clinic by community health workers. At the clinics, we randomly selected intake forms from the Program for Family Health for age and sex at each clinic and contacted those individuals for inclusion in the control group. Each of the matched controls were clinically evaluated for signs of leprosy. Any individual with a clinical suspicion of leprosy was excluded from the study and referred to municipal health centers for further diagnostic testing.

### Data collection

Data collection was conducted between April and August 2010. Data collection was coordinated through the Municipality Health Secretariats with the support of the Maranhão State Health Secretariat. Study participants were recruited by community health agents for the study. They were interviewed by trained health professionals at the local health care centers, or in patient homes when disability or age prevented health center attendance. Information on demographics, socioeconomic status, healthcare access, migration, behavior and stress was collected through structured questionnaires. Clinical data were also collected through patient medical records.

### Data analysis

Data were entered twice using EpiInfo software version 3.5.1 (Centers for Disease Control and Prevention, Atlanta, USA) and cross-checked for entry-related errors. Statistical tests were used to assess normality. Included are data sets with information related to migration. Any cases that did not have complete migration data were excluded from the analysis. Of the 340 leprosy cases and 340 matched controls, we first identified 135 (19.9%) past 5-year migrants in the case (n = 80) and control groups (n = 55). The distribution of key demographic, spatial and temporal migration pattern variables among past 5-year migrants in the case and control groups was examined and tested by the use of Fishers exact test for significant differences in the stratified sample of past 5-year migrants.

We then conducted bivariate analyses comparing cases (n = 340) and controls (n = 340) using Fishers exact test to examine if past five year migration was associated with leprosy diagnosis. As household contact remains the most significant known transmission risk to date for leprosy infection [14], [15], we additionally undertook multivariate logistic regression analysis controlling for family (parent, child and/or sibling) and household (consanguineous and/or non-consanguineous) contact with leprosy.

Next, stratified bivariate analyses using Fishers exact tests were used to determine differences in the association among social determinants of health (socioeconomic status), psychosocial (alcohol use and life stressors) and biosocial factors (leprosy contact exposure) for case and control groups of past 5-year migrants (n = 135).

## Results

**Figure 1:**
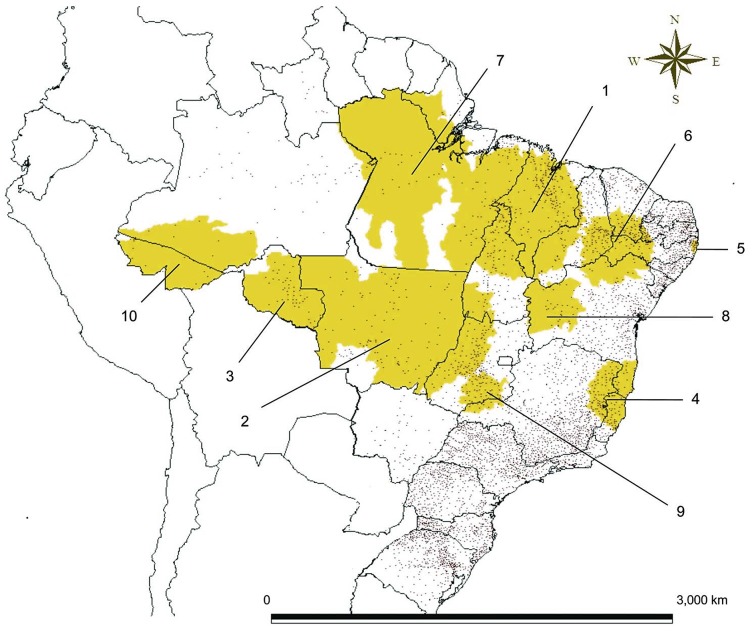
Locations of the 10 most probable leprosy clusters (yellow regions) and municipal councils (dots), Brazil, 2005–2007. [6].

A total of 394 leprosy cases and 391 controls were interviewed. There were 23 relapsed leprosy cases and 12 controls suspected of leprosy who were excluded from the study. Eight respondents refused to participate. Complete migration data was available for 680 respondents. Of the 340 leprosy cases and 340 matched clinically unapparent controls, 23.5% of those with leprosy (n = 80 cases) and 16.2% (n = 55) of the control group without clinical signs of leprosy migrated in the past 5 years before diagnosis. Only 4.4% (n = 15) of cases migrated after diagnosis. Table 1 reflects migration into and out of major endemic clusters identified by the Brazilian Ministry of Health as high-risk areas for leprosy transmission [6] (Figure 1), and other demographics and migration variables. These variables were not significantly associated with leprosy among past 5-year migrants prior to diagnosis (test results not shown). Leprosy cases were largely among the youngest age group (15–29) migrating, with an equal distribution between males and females. More than one-third of those with leprosy who migrated in the past five years were illiterate. The majority of leprosy cases migrated within cluster 1, which includes the northern states of Pará, Piauí, Tocantins and Maranhão. More than half (56.3%) of cases moved between municipalities in Maranhão, followed with fewer cases to neighboring Pará (11.8%), Piauí (3.9%) and Tocantins (2.0%), and one-fifth of migrants were drawn to non-contiguous states. All those with leprosy migrated into a highly endemic cluster on at least one occasion, not including their current residence.

**Table 1:** Demographics and migration patterns of past 5-year migrant leprosy cases and clinically unapparent controls.

	Leprosy Cases		Controls	
	Included*(n = 80)†	%	Included (n = 55)†	%
**Demographics**				
Age				
15–29	35	43.8	28	50.9
30–44	21	26.3	14	25.5
45–59	15	18.8	9	16.4
60 or older	9	11.3	4	7.3
Gender				
Male	40	50.0	35	63.6
Female	40	50.0	20	36.4
Education				
Literate	54	67.5	45	81.8
Illiterate	26	32.5	10	18.2
**Migration Patterns**				
Leprosy Cluster Migration				
Cluster 1	48	60.0	32	58.2
Cluster 2	3	3.8	1	1.8
Cluster 6	1	1.3	0	0
Cluster 7	1	1.3	1	1.8
Cluster 9	2	2.5	0	0
Out of cluster migration	25	31.3	21	38.2
Migration in cluster				
1 time	71	88.8	49	89.1
2 or more times	9	11.3	5	9.1
In-state vs. out of state migration				
In Maranhão	45	56.3	25	45.5
Other state	35	43.75	30	54.6
No. of times migrated past 5-yrs				
1 time	61	76.3	47	85.5
2 or more times	19	23.8	8	14.5
Zone of migration in past 5-yrs				
Urban only	47	60.3	38	70.4
Rural only	26	33.3	13	24.1
Rural and Urban	6	7.7	3	5.6
Migration for work in past 5-yrs				
Yes	46	57.5	30	55.6
No	34	42.5	25	45.5
Social network prior to migration				
Always	63	79.8	39	70.9
Sometimes	5	6.3	1	1.8
Never	11	13.9	15	27.3
Who lived with during migration				
Family	64	81.0	41	74.6
Co-workers	14	17.7	12	21.8
Other	1	1.3	2	3.6
Mean # people lived with during migration	80	8.61	55	6.7
Mean years of migration		6.25		4.8

† Data not available for all individuals.

Nearly one in six migrants with leprosy migrated for employment in the last five years and this was slightly less than expected for internal population movement. Typical of internal population flow, most migration in Maranhão was to urban areas (60.3%) compared to rural areas (33.3%), and both rural and urban areas (7.7%). Social networks in migration destination sites for those with leprosy had a higher tendency to be family contacts with whom they lived (81.0%) than work contacts (17.7%). This may be an explanation for the significant number of respondents who always had a contact prior to migrating (79.8%). Migrants with leprosy lived on average with 8.61 people per household while migrating.

Past five year migration prior to diagnosis was found to be significantly associated with leprosy as shown in Table 2 which represents the results of the multivariate logistic regression analysis. Past five year migration remained significantly associated with leprosy after controlling in separate models for 1) household contact (consanguineous and/or non-consanguineous); 2) family contact (parent, child and/or sibling; 3) and household and family contact in multiple logistic regression models.

**Table 2:** Crude (OR) and adjusted odds ratios (AOR) for the association of leprosy and five year migration prior to leprosy diagnosis, controlling for household, family, and household and family leprosy contact.

					AOR Controlling for leprosy contact		
	Included (n = 680)	Leprosy Cases N (%)	Controls N (%)	OR (95% CI)	Household contact	Family contact	Household/Family contact
**Past five year migration**							
**Yes**	135	80 (59.3)	55 (40.7)	1.59 (1.07–2.38)*	1.54 (1.03–2.29)*	1.51 (1.01–2.27)*	1.51 (1.0–2.28)**
**No**	545	260 (47.7)	285 (52.3)	1.0	1.0	1.0	1.0

* P<.05.

** P<.10.

Key social, biosocial, and behavioral factors were found to be associated with leprosy (Table 3). Household, familial and other contact with someone infected with leprosy was significantly different for leprosy infected past 5-year migrants compared to control group migrants. Genetic association of closely related kinship shows a significant difference for contact with parent/child/sibling (OR: 7.82; CI 95%: 2.32–33.38; P-value = 0.0001). Contact regardless of consanguinity (OR: 4.99; CI 95%: 1.7–16.51; P-value = 0.001) and actual household contact (OR: 5.54; CI 95%: 1.49–30.46; P-value = 0.004) was also significant. An important behavioral factor distinguishing migrants with leprosy compared to the clinically unapparent control group was past five year alcohol consumption (OR: 4.46; CI 95%: 1.43–14.15; P-value = 0.005).

**Table 3:** Factors associated with leprosy diagnosis among past five year migrant cases and clinically unapparent controls.

Social and Behavioral Variables	Included (n = 135) ††	Leprosy Cases N (%)	Controls N (%)	OR (95% CI)	P-value
**Alcohol consumption**					
Never drank	29	15 (51.72)	14 (48.28)	Reference	
Drink currently	43	15 (34.88)	28 (65.12)	0.5 (0.17–1.45)	0.22
Drank in past 5 years	52	43 (82.69)	9 (17.31)	4.46 (1.43–14.15)	0.005
Stopped drinking more than 5 years ago	11	7 (63.64)	4 (36.36)	1.63 (0.32–9.25)	0.72
**Leprosy Contact**					
Familial and non-familial contact					
No leprosy contact	76	33 (43.42)	43 (56.58)	Reference	
Parent/Child/Sibling with leprosy	28	24 (85.71)	4 (14.29)	7.82 (2.32–33.38)	0.0001
Others with leprosy	29	23 (79.31)	6 (20.69)	4.99 (1.7–16.51)	0.001
Household contact with leprosy past 5/6 years					
Yes	23	20 (86.96)	3 (13.04)	5.54 (1.49–30.46)	0.004
No	108	59 (54.63)	49 (45.37)	Reference	
**Socio-economic factors**					
Income†					
< = R$510	55	38 (69.09)	17 (30.91)	2.12 (0.97–4.71)	0.049
>R$510	76	39 (51.32)	37 (48.68)	Reference	
Public Waste Service					
Yes	107	58 (54.21)	49 (45.79)	Reference	0.03
No	28	22 (78.57)	6 (21.43)	3.1 (1.1–10.02)	
Family Illiteracy					
Yes	44	32 (72.73)	12 (27.27)	2.67 (1.13–6.51)	
No	78	39 (50.0)	39 (50.0)	Reference	0.02

† At the time of the survey 1US$ was equivalent to 1.72R$, and R$ 511,- the official minimum wage as set by the Federal Government.

†† Data not available for all individuals.

Income and other socioeconomic variables showed significant differences between migrants with leprosy and the control group. Income less than the minimum wage (OR: 2.12; CI 95%: 0.97–4.71; p-value = 0.049) as well as poor access to public waste services (OR: 3.1; CI 95%: 1.1–10.02; p-value = 0.03) and family illiteracy (OR: 2.67; CI 95%: 1.13–6.51; p-value = 0.02) were found to be associated with leprosy among past 5-year migrants.

Education, presence of BCG scar, zone of residence and lifestyle stressors - separation from family and friends, loss of employment or income, marital separation or death of close friend or relative- were not significantly associated with leprosy among past five year migrants.

## Discussion

Leprosy was introduced to Brazil through European colonization and later through slave movement so that by the 1600's, leprosy was well established in the country [25]. More recently, migration has been hypothesized to be an impediment to leprosy control, and spatial analysis indicates the introduction of leprosy through inter and intra-state population movement in Brazil [5], as well as expanded distribution of leprosy through migration [26]. Population movement can put both migrants and non-migrants at risk when diseases move between endemic and non-endemic areas. Latent symptomology, characteristic of leprosy, could facilitate the distribution of disease when no symptoms are present, or when mild symptoms are overlooked. The migrant lifestyle poses similar marginalized socioeconomic, behavioral and environmental risks that have been well established as factors associated with leprosy transmission [4], [10]–[13], [27]–[28].

Leprosy in this study was found to be significantly associated with past five year migration. Susceptibility among migrants may, in part, be due to spatial and temporal patterns of movement in and between areas identified by the Brazilian Ministry of Health as highly endemic clusters for leprosy transmission [16]. While we found no significant difference between key spatial and temporal variables and past five year migration among those with leprosy compared to the clinically unapparent control group, more than half of movement for internal migration among those with leprosy was within the leprosy endemic cluster in the state of Maranhão. Few migrated to the other nine endemic clusters in Brazil, a third migrated to other non-endemic areas, and less than half migrated outside of Maranhão. From an operational perspective for leprosy control in Brazil, this provides sufficient evidence to suggest future surveillance of population flow between municipalities in Maranhão, which should involve comparison of the distribution of leprosy incidence over the five year latency period. Should these areas be identified as emerging endemic areas, service delivery strategies should target these as focal points for state control efforts.

Maranhão continues to be a state with higher net out- and return- migration [9]. Interstate population movement, such as to neighboring Pará, draws many poor migrants from Maranhão's interior leprosy endemic areas to the employment found in large-scale mining and agriculture industries [29]–[30]. Interstate movement necessitates cross-border cooperation for leprosy control and may aid in identifying impending high-risk areas for disease distribution. In fact, other research showed that 5.2% of leprosy patients in *Cluster 1* (including Maranhão and neighboring states of Tocantins, Piaui and Pará) were diagnosed outside of their municipality of residence between 2001–2009 [31]. Municipalities in Maranhão and neighboring Pará, which have the third and fifth highest new case incidence in the country respectively, would be good targets for future collaborative surveillance projects.

Our findings indicate that the majority of migration in Maranhão continues to be between rural and urban areas, consistent with other research on population flow in Brazil [32]. However population movement documented in our study appears to be of longer duration than is typical for temporary circular migration. Rural to urban migration is a common solution to reduce poverty, as more and regular job opportunities tend to exist in urban areas [33]–[35]. This often places migrants at higher risk for disease morbidity and mortality due to poor living conditions in urban slums [36]. Kerr-Pontes et al.'s (2004) [4] ecological study in Brazil's northeast demonstrated that urban population growth due to uncontrolled urbanization and migrant influx from Brazil's rural interior, was a predictor of leprosy incidence.

We found that population movement is clearly facilitated through strong destination based social networks as a precursor to migration. These social networks tend to be family-based, as indicated by migrant co-habitation arrangements. On an individual level, social networks enable population movement by reducing the cost of migration through benefits such as established shared housing and employment networks, thus making migration a more attractive option to pursue. On a community level, social networks that facilitate migration can have a cumulative effect in sending municipalities to perpetuate and build upon migrant flow between origin and destination sites [37]. Because of the social nature of these community relationships to kinship, friendships and working relationships, migration can be highly localized to movement between specific neighborhoods in sending and receiving communities.

Short-term movement, as Skeldon (2003) [38] points out, is less likely to be measured through census surveys, thus monitoring population movement should be undertaken at the municipality level and integrated into larger databases to establish early warning systems.

Exposure to an index patient has been identified as the primary determinant of leprosy infection among their contacts. The magnitude of the effect of contact in our study was highest among close family contact – parent, child, and/or siblings - followed by consanguineous and/or non-consanguineous household contact and lastly other contact, which could include social and distant family exposure. The possibility of genetic susceptibility to leprosy infection, through close family kinship has been significantly associated with leprosy among contacts [14]–[15], [39] which supports our findings of leprosy association with close kinship among past 5-year migrants. At the household level, other research has shown that proximity to and intensity of exposure to leprosy increases the risk of transmission, as much as five to nine times that of non-household contacts [14]–[15], [39]–[42], although leprosy clustering among neighboring residences in areas of high population density and poverty has social contact risk similar to household contacts [43]. Contact with multibacillary diagnosis in the household has also been associated with increased risk [14]–[15], [41]–[42] and indicates late diagnosis and long-term exposure to contacts. As the majority of migrants in our sample were diagnosed with multibacillary leprosy, this has significant implications for transmission and also for leprosy associated complications and disability.

Migration was significantly associated with leprosy in our logistic regression models controlling for household and close family contact independently. The independent association with household and close consanguineous exposure could indicate some relationship to familial social networks in migrant destination sites. This, in addition to intensity of exposure due to high household density during migration, suggests both the genetic relationships and social environment surrounding migration may figure prominently in explaining leprosy diagnosis.

The majority of individuals in contact with an index patient are not susceptible to the disease. As such, Sales et al. (2011) [14] suggest that leprosy surveillance should explore multiple factors that may contribute to the risk for infection. While many behavioral, demographic, and socio-environmental variables were included in the analysis, we found socioeconomic status and past five year alcohol consumption among migrants with leprosy were significantly associated with leprosy in comparison to clinically unapparent migrants in the control group. Brazil has one of the highest alcohol-attributable disability-adjusted life years (DALYs) in the world. According to the World Health Organization, there is evidence for an association between alcohol consumption and infectious disease [44]. Current alcohol use however was not significant. This may be the result of recently diagnosed migrants abstaining from alcohol use due to multi-drug therapy treatment. A substantial concern, however was that nearly one in five migrants with leprosy were currently drinking alcohol, which has been associated with leprosy relapse in Brazil [12]. Alcohol consumption can interact with medication absorption [45] and could render leprosy treatment less effective. This can contribute to the elevation of risk for transmission to exposed contacts.

Low socioeconomic status was additionally associated with leprosy among past 5-year migrants. Other research in *Cluster 1* also found poverty associated with migration prior to diagnosis among those with leprosy (unpublished data). While poverty is ubiquitously associated with leprosy throughout the literature, it should be noted that these results were taken after the migration period and thus may not be an adequate measure of socioeconomic level during migration. Low socioeconomic status among migrants with leprosy may be linked to restricted employment as the result of disability due to leprosy, or difficulty in sustaining employment during treatment. Despite this, family illiteracy and inaccessibility to public waste collection, proxies for low socioeconomic status in Brazil, were significantly higher for migrants with leprosy compared to the control group. Socioeconomic status, the primary social determinant of health, should be the topic of further investigation both during and after migration.

### Conclusion

Leprosy was found to be associated with past five year migration, even after controlling for confounders. In the comparison of past 5-year migrants, leprosy was associated with both household consanguineous and/or non-consanguineous contact, close family and other social leprosy contact, consistent with research identifying contact exposure as the major determinant of leprosy transmission [14]–[15]. However, the magnitude of effect for leprosy among migrants in our study was most significant among close family and household contacts. As migration in Maranhão was largely facilitated through family networks, contact surveillance should include migration site residence contacts as well as current residence contacts.

While patterns of migration, including movement in and between highly endemic clusters, were not different among migrants with leprosy and clinically unapparent migrants in the control group, important facets of migration emerged that could benefit leprosy control at the state and national level. State control programs should consider monitoring past five year residence among those newly diagnosed with leprosy to identify intra- and inter-state migration flow. This may provide early warning systems for localized disease control in areas yet to be identified as high-risk areas.

Alcohol consumption in the years prior to diagnosis may be associated with susceptibility to leprosy. Alcohol consumption and consumption frequency should be included in future investigations. This research will help to determine the extent that alcohol consumption plays a role in the dynamics of both transmission and expression of leprosy. As alcohol consumption has also been associated with leprosy relapse, further attention should be given to alcohol consumption during treatment, patient relapse and contact exposure to leprosy. Other substances should also be given attention in future research.

Other research in Brazil has found a spatial relationship to migration and distribution of leprosy and an association of leprosy with poor socio-economic conditions [4]–[6]. Our research shows that in endemic areas leprosy is not only associated with population movement itself, but, most importantly with the social conditions of the migrant in the endemic areas, their behavior, and contact with leprosy in the family and household.

## Supporting Information

Checklist S1 STROBE checklist.
(DOC)
